# Algebraic Quantitative Semantics for Efficient Online Temporal Monitoring

**DOI:** 10.1007/978-3-030-72016-2_18

**Published:** 2021-03-01

**Authors:** Konstantinos Mamouras, Agnishom Chattopadhyay, Zhifu Wang

**Affiliations:** 8 Eindhoven University of Technology, Eindhoven, The Netherlands; 9 Aalborg University, Aalborg East, Denmark; Rice University, Houston, TX 77005 USA

**Keywords:** Online Monitoring, Verification, Quantitative Semantics.

## Abstract

We investigate efficient algorithms for the online monitoring of properties written in metric temporal logic (MTL). We employ an abstract algebraic semantics based on semirings. It encompasses the Boolean semantics and a quantitative semantics capturing the robustness of satisfaction, which is based on the max-min semiring over the extended real numbers. We provide a precise equational characterization of the class of semirings for which our semantics can be viewed as an approximation to an alternative semantics that quantifies the distance of a system trace from the set of all traces that satisfy the desired property.

### 1 Introduction

Online monitoring is a lightweight verification technique for checking during runtime that a system behaves as desired. It has proved to be effective for evaluating the correctness of the behavior of complex systems, which includes cyber-physical systems (CPSs) that consist of both computational and physical processes. An *online monitor* is a program that observes the execution trace of the system and emits values that indicate events of interest or other actionable information.

It is common to specify monitors using special-purpose formalisms such as variants of temporal logic and domain-specific programming languages. In the context of cyber-physical systems, logics that are interpreted over signals are frequently used. This includes Metric Temporal Logic (MTL) [30] and Signal Temporal Logic (STL) [33]. We focus here on properties specified with MTL and interpreted over discrete-time signals. We do not restrict the outputs of the monitor to Boolean (qualitative) verdicts, but allow for a quantitative interpretation of property satisfaction that admits various degrees of truth or falsity. Such quantitative interpretations of temporal logic have been considered before, including several variants of the so-called robust semantics of MTL [22,20,5].

Our starting point is the widely-used spatial robust semantics of MTL [22]. This uses the set $\mathbb{R}^{\pm\infty} = \mathbb{R} \cup \{-\infty, \infty\}$ of the extended real numbers as truth values, where a positive number indicates truth, a negative number indicates falsity, and zero is ambiguous. Disjunction is interpreted as max, and conjunction is interpreted as min. Two quantitative semantic notions are considered in [22]:

(1) the *robustness degree* $\mathsf{degree}(\varphi, u)$ of a trace $u$ w.r.t. a formula $\varphi$, which is defined in a global way using distances between signals, and (2) the *robust semantics* $\rho(\varphi, u)$ of a formula $\varphi$ w.r.t. a trace $u$, which is defined by induction on the structure of $\varphi$. The former notion is the primary definition that captures the intuitive idea of the degree of satisfaction, whereas the latter is used as an approximate estimate. The usefulness of this estimate is justified by establishing a precise relationship between the two values [22]. The robust semantics of [22] has been used in prior work on online monitoring [16,15].

We embark on an investigation of how to generalize the robustness framework of [22] to other notions of quantitative truth values. Instead of focusing exclusively on the concrete structure $(\mathbb{R}^{\pm\infty}, \sup, \inf, -\infty, \infty)$, we take an *abstract algebraic* approach and look at classes of structures that are defined axiomatically. We start by considering the class of semirings, algebraic structures of the form $(V, +, \cdot, 0, 1)$ with an addition operation $+$ (which models disjunction) and a multiplication operation $\cdot$ (which models conjunction) satisfying a set of equational laws. The class of semirings contains $\mathbb{B} = \{\bot, \top\}$ (the Boolean values), $(\mathbb{R}^{\pm\infty}, \max, \min, -\infty, \infty)$, the max-plus (tropical) semiring $(\mathbb{R} \cup \{-\infty\}, \max, +, -\infty, 0)$, and $(\mathbb{R}, +, \cdot, 0, 1)$. The semiring of intervals with (semiring) addition given by $[a, b] \oplus [c, d] = [\max(a, c), \max(b, d)]$ and (semiring) multiplication given by $[a, b] \otimes [c, d] = [\min(a, c), \min(b, d)]$ is an especially interesting example, as it can be used to model uncertainty in the truth value: an element $[a, b]$ indicates that the truth value lies somewhere within this interval.

We use an algebraic generalization of the inductively-defined robust semantics of [22], as our goal is to obtain online monitors that are time- and space-efficient. Our main results are the following:

- The theorem of [22] that relates $\mathsf{degree}(\varphi, u)$ and $\rho(\varphi, u)$ is generalized from $\mathbb{R}^{\pm\infty}$ to a class of semirings. The class of semirings for which the theorem holds admits a precise axiomatic characterization (Theorem 7). To obtain this, we develop a notion of symbolic quantitative languages that forms a semantic bridge between quantitative specifications and sets of traces.
- We propose a new algorithm for efficient online monitoring (Theorem 11) that goes beyond existing algorithms. Prior monitors [16,15] compute max or min over sliding-windows and therefore apply only to semirings that are linear orders (e.g., $\mathbb{B}$ and $\mathbb{R}^{\pm\infty}$). Our monitoring algorithm applies to values $V$ that are partial orders or more general semirings. In order to obtain this algorithm, we reduce the monitoring of formulas of the form $\varphi \, \mathsf{S}_{[a,b]} \, \psi$ and $\varphi \, \mathsf{U}_{[a,b]} \, \psi$ to a sliding-window aggregation (which is neither max nor min).

We provide an implementation of our algebraic monitoring framework in Rust. Our experiments show that our monitors scale reasonably well and they compare favorably against the state-of-the-art monitoring tool Reelay [40].

### 2 Algebraic Semantics using Semirings

A *semiring* is an algebraic structure $(V, +, \cdot, 0, 1)$, where $+$ is called addition and $\cdot$ is called multiplication, that satisfies the following properties: (1) $(V, +, 0)$ is a commutative monoid, (2) $(V, \cdot, 1)$ is a monoid, (3) multiplication distributes over addition, and (4) 0 is an annihilator for multiplication. The last two properties say that $x(y+z) = xy+xz$, $(x+y)z = xz+yz$, and $0x = x0 = 0$ for all $x, y, z \in V$. We sometimes write $xy$ to mean $x \cdot y$. A semiring $V$ is called *idempotent* if addition is idempotent, that is, $x + x = x$ for every $x \in V$. For an idempotent semiring, we define the partial order induced by $+$ as follows: $x \leq y$ iff $x + y = y$. A *homomorphism* from a semiring $U$ to a semiring $V$ is a function $h : U \to V$ that commutes with the semiring operations. An *epimorphism* is a surjective homomorphism. Let $U$ and $V$ be idempotent semirings and $h : U \to V$ be a semiring homomorphism. Then, $h$ is *monotone* (i.e., *order-preserving*).

**Example 1.** The set $\mathbb{B} = \{\bot, \top\}$ of Boolean values with disjunction and conjuction is a semiring. The set $\mathbb{T} = \{\bot, ?, \top\}$ can be endowed with semiring structure as follows: $x + \bot = x$, $x + \top = \top$, $? + ? = ?$, $x \cdot \bot = \bot$, $x \cdot \top = x$, and $? \cdot ? = ?$, where $\cdot$ is commutative. The structure $\mathbb{T}$ is used to give a *three-valued* interpretation of formulas (? is inconclusive). The structure $(\mathbb{R}^{\pm\infty}, \max, \min, -\infty, \infty)$ is the *max-min semiring* over the extended reals. The structure $(\mathbb{R}, +, \cdot, 0, 1)$ is a semiring and $\mathbb{Z}$ (integers) and $\mathbb{N}$ (natural numbers) are subsemirings of it.

We interpret the max-min semiring $\mathbb{R}^{\pm\infty}$ as degrees of truth, where positive means true and negative means false. The value 0 is ambiguous. For this reason we also consider a variant of $\mathbb{R}^{\pm\infty}$, where the value 0 is refined into a positive $+0$ (true) and a negative $-0$ (false). We thus obtain the max-min semiring $\mathbb{R}^{\pm\infty}_{\pm 0}$, which is isomorphic to $\mathbb{B} \times \mathbb{R}_{\geq 0}$, where $\mathbb{R}_{\geq 0} = \{x \in \mathbb{R} \mid x \geq 0\}$.

For integers $i, j \in \mathbb{Z}$ we define the intervals $[i, j] = \{n \in \mathbb{Z} \mid i \leq n \leq j\}$ and $[i, \infty) = \{n \in \mathbb{Z} \mid i \leq n\}$. For a set $I$ of integers and $n \in \mathbb{Z}$, define $n + I = \{n + i \mid i \in I\}$ and $n - I = \{n - i \mid i \in I\}$.

For a semiring $V$, an interval $I = [i, j]$ (where $i, j$ are natural numbers) and an $I$-indexed tuple $\bar{x} = (x_i)_{i \in I}$ whose components are in $V$, we define $\sum \bar{x} = \sum_{k \in I} x_k = \sum_{k=i}^{j} x_k = x_i + \cdots + x_j$ and $\prod \bar{x} = \prod_{k \in I} x_k = \prod_{k=i}^{j} x_k = x_i \cdots x_j$. If the tuple $\bar{x}$ is empty (i.e., $I = \emptyset$) then we define $\sum \bar{x} = 0$ and $\prod \bar{x} = 1$.

We will consider formulas of Metric Temporal Logic (MTL) interpreted over *traces* that are finite or infinite sequences of *data items* from a set $D$. We write $D^*$ (resp., $D^+$) for the set of all finite (resp., non-empty finite) sequences over $D$, and $D^\omega = \omega \to D$ for the set of all infinite sequences over $D$, where $\omega$ is the first infinite ordinal (i.e., the set of natural numbers). We also define $D^\infty = D^* \cup D^\omega$. We write $\varepsilon$ for the empty sequence and $|u|$ for the length of a trace, where $|u| = \omega$ if $u$ is infinite. A finite sequence $u \in D^*$ can be viewed as a function from $\{0, \ldots, |u| - 1\}$ to $D$, that is, $u = u(0)u(1) \ldots u(|u| - 1)$. We also consider a semiring $V$ whose elements represent quantitative truth values, and *unary quantitative predicates* $p : D \to V$. We write $\mathbb{1}, \mathbb{0} : D \to V$ for the predicates given by $\mathbb{1}(d) = 1$ and $\mathbb{0}(d) = 0$ for every $d \in D$.

The set $\mathsf{MTL}(D, V)$ of ***temporal formulas*** is built from the atomic predicates $p : D \to V$ using the Boolean connectives $\vee$ and $\wedge$, the unary temporal connectives $\mathsf{P}_I$, $\mathsf{H}_I$, $\mathsf{F}_I$, $\mathsf{G}_I$, and the binary temporal connectives $\mathsf{S}_I$, $\bar{\mathsf{S}}_I$, $\mathsf{U}_I$, $\bar{\mathsf{U}}_I$, where $I$ is an interval of the form $[i, j]$ or $[i, \infty)$ with $i, j < \omega$. For every temporal

$$\rho(p, u, i) = p(u(i))$$

$$\rho(\varphi \vee \psi, u, i) = \rho(\varphi, u, i) + \rho(\psi, u, i) \qquad \rho(\varphi \wedge \psi, u, i) = \rho(\varphi, u, i) \cdot \rho(\psi, u, i)$$

$$\rho(\mathsf{P}_I \varphi, u, i) = \textstyle\sum_{j \in i-I,\, j \geq 0} \rho(\varphi, u, j) \qquad \rho(\mathsf{H}_I \varphi, u, i) = \textstyle\prod_{j \in i-I,\, j \geq 0} \rho(\varphi, u, j)$$

$$\rho(\mathsf{F}_I \varphi, u, i) = \textstyle\sum_{j \in i+I,\, j < |u|} \rho(\varphi, u, j) \qquad \rho(\mathsf{G}_I \varphi, u, i) = \textstyle\prod_{j \in i+I,\, j < |u|} \rho(\varphi, u, j)$$

$$\rho(\varphi \,\mathsf{S}_I\, \psi, u, i) = \textstyle\sum_{j \in i-I,\, j \geq 0} \Big( \rho(\psi, u, j) \cdot \prod_{k=j+1}^{i} \rho(\varphi, u, k) \Big)$$

$$\rho(\varphi \,\bar{\mathsf{S}}_I\, \psi, u, i) = \textstyle\prod_{j \in i-I,\, j \geq 0} \Big( \rho(\psi, u, j) + \sum_{k=j+1}^{i} \rho(\varphi, u, k) \Big)$$

$$\rho(\varphi \,\mathsf{U}_I\, \psi, u, i) = \textstyle\sum_{j \in i+I,\, j < |u|} \Big( \prod_{k=i}^{j-1} \rho(\varphi, u, k) \Big) \cdot \rho(\psi, u, j)$$

$$\rho(\varphi \,\bar{\mathsf{U}}_I\, \psi, u, i) = \textstyle\prod_{j \in i+I,\, j < |u|} \Big( \sum_{k=i}^{j-1} \rho(\varphi, u, k) + \rho(\psi, u, j) \Big)$$

Fig. 1: Semiring-based quantitative semantics for MTL.

connective $X \in \{\mathsf{P}, \mathsf{H}, \mathsf{S}, \bar{\mathsf{S}}, \mathsf{F}, \mathsf{G}, \mathsf{U}, \bar{\mathsf{U}}\}$, we write $X_i$ as an abbreviation for $X_{[i,i]}$ and $X$ as an abbreviation for $X_{[0,\infty)}$.

Since we focus in this paper on online monitoring, we restrict attention to the ***future-bounded*** fragment of $\mathsf{MTL}$, where the future-time temporal connectives are bounded. That is, every $\mathsf{U}_I$ connective is of the form $\mathsf{U}_{[a,b]}$ for $a \leq b < \omega$ (and similarly for $\mathsf{F}_I$, $\mathsf{G}_I$, $\bar{\mathsf{U}}_I$). We always assume this restriction on formulas.

We interpret the formulas in $\mathsf{MTL}(D, V)$ over traces from $D^\infty$ and at specific time points. The *interpretation function* $\rho : \mathsf{MTL}(D, V) \times D^\infty \times \omega \to V$, where $\rho(\varphi, u, i)$ is defined when $i < |u|$, is shown in Fig. 1. We say that the formulas $\varphi$ and $\psi$ are *equivalent*, and we write $\varphi \equiv \psi$, if $\rho(\varphi, u, i) = \rho(\psi, u, i)$ for every $u \in D^\infty$ and $i < |u|$. For every formula $\varphi$ and every interval $I$, it holds that $\mathsf{P}_I \varphi \equiv \mathbb{1} \,\mathsf{S}_I\, \varphi$, $\mathsf{H}_I \varphi \equiv \mathbb{0} \,\bar{\mathsf{S}}_I\, \varphi$, $\mathsf{F}_I \varphi \equiv \mathbb{1} \,\mathsf{U}_I\, \varphi$, and $\mathsf{G}_I \varphi \equiv \mathbb{0} \,\bar{\mathsf{U}}_I\, \varphi$.

We say that a semiring $V$ *refines* $\mathbb{B}$ if there is a semiring homomorphism $h : V \to \mathbb{B}$. Notice that $h$ is necessarily an epimorphism because $h(0) = \bot$ and $h(1) = \top$. Informally, we think of $h^{-1}(\bot)$ as the subset of "false" values and $h^{-1}(\top)$ as the subset of "true" values. In particular, this means that $V$ can be partitioned into true and false values. There are semirings that cannot refine $\mathbb{B}$. For example, the semiring $(\mathbb{Z}, +, \cdot, 0, 1)$ of the integers cannot refine $\mathbb{B}$.

Let $h : V \to \mathbb{B}$. For a predicate $p : D \to V$, we say that $d \in D$ *$h$-satisfies* $p$, and we write $d \models_h p$, if $h(p(d)) = \top$. For $u \in D^\infty$ and $i < |u|$ we define the *satisfaction relation* $\models_h$ as usual (for atomic formulas: $u, i \models_h p$ iff $u(i) \models_h p$).

**Lemma 2.** Let $D$ be a set of data items, $V$ be a semiring, and $h : V \to \mathbb{B}$. The following are equivalent:
(1) The function $h$ is a semiring homomorphism.
(2) $u, i \models_h \varphi$ iff $h(\rho(\varphi, u, i)) = \top$ for every $\varphi : \mathsf{MTL}(D, V)$, $u \in D^\infty$ and $i < |u|$.

Lemma 2 says that the qualitative semantics $\models_h$ agrees with the quantitative semantics $\rho$ exactly when $h : V \to \mathbb{B}$ is a semiring homomorphism. In this case, $\rho$ is more fine-grained and loses no information regarding Boolean satisfaction.

**Lemma 3.** Let $D$ be a set of data items and $V$ be a semiring. The identities of Fig. 2 hold for all formulas $\varphi, \psi \in \mathsf{MTL}(D, V)$.

$$
\begin{aligned}
\mathsf{P}\varphi &\equiv \mathsf{P}_1(\mathsf{P}\varphi) \vee \varphi \quad\text{and}\quad \mathsf{H}\varphi \equiv \mathsf{H}_1(\mathsf{H}\varphi) \wedge \varphi \\
\varphi\,\mathsf{S}\,\psi &\equiv (\mathsf{P}_1(\varphi\,\mathsf{S}\,\psi) \wedge \varphi) \vee \psi \\
\mathsf{P}_{[a,\infty)}\varphi &\equiv \mathsf{P}_a\mathsf{P}\varphi \quad\text{and}\quad \mathsf{H}_{[a,\infty)}\varphi \equiv \mathsf{H}_a\mathsf{H}\varphi \\
\varphi\,\mathsf{S}_{[a,\infty)}\,\psi &\equiv \mathsf{P}_a(\varphi\,\mathsf{S}\,\psi) \wedge \mathsf{H}_{[0,a-1]}\varphi, \ \text{for } a \geq 1 \\
\mathsf{P}_{[a,b]}\varphi &\equiv \mathsf{P}_a\mathsf{P}_{[0,b-a]}\varphi \quad\text{and}\quad \mathsf{H}_{[a,b]}\varphi \equiv \mathsf{H}_a\mathsf{H}_{[0,b-a]}\varphi \\
\varphi\,\mathsf{S}_{[a,b]}\,\psi &\equiv \mathsf{P}_a(\varphi\,\mathsf{S}_{[0,b-a]}\,\psi) \wedge \mathsf{H}_{[0,a-1]}\varphi, \ \text{for } a \geq 1 \\
\mathsf{F}_{[0,b]}\varphi &\equiv \mathsf{F}_b\mathsf{P}_{[0,b]}\varphi \quad\text{and}\quad \mathsf{G}_{[0,b]}\varphi = \mathsf{G}_b\mathsf{H}_{[0,b]}\varphi \\
\mathsf{F}_{[a,b]}\varphi &\equiv \mathsf{F}_b\mathsf{P}_{[0,b-a]}\varphi \quad\text{and}\quad \mathsf{G}_{[a,b]}\varphi \equiv \mathsf{G}_b\mathsf{H}_{[0,b-a]}\varphi \\
\varphi\,\mathsf{U}_{[a,b]}\,\psi &\equiv \mathsf{G}_{[0,a-1]}\varphi \wedge \mathsf{F}_a(\varphi\,\mathsf{U}_{[0,b-a]}\,\psi), \ \text{for } a \geq 1
\end{aligned}
$$

Fig. 2: Equivalences between temporal formulas.

The identities of Fig. 2 are all shown using the semiring axioms. The identity below can be used to reduce the monitoring of $\mathsf{S}_{[0,a]}$ to $\mathsf{P}_{[0,a]}$.

$$\varphi\,\mathsf{S}_{[0,a]}\,\psi \equiv (\varphi\,\mathsf{S}\,\psi) \wedge \mathsf{P}_{[0,a]}\psi \tag{1}$$

An early occurrence of this idea is in [19], where they consider the more general (future-time) form $\varphi\,\mathsf{U}_{[a,b]}\,\psi \equiv (\varphi\,\mathsf{U}_{[a,\infty)}\,\psi) \wedge \mathsf{F}_{[a,b]}\psi$. Prior work on efficient monitoring [15] uses an algorithm based on it. Specifically, [15] uses a sliding-max algorithm [32], which can be applied to the max-min semiring $\mathbb{R}^{\pm\infty}$ and other similar linear orders, but is not applicable to partial orders or other semirings.

**Proposition 4.** For a set $D$ with at least two elements and a semiring $V$, the following are equivalent:
(1) The semiring $V$ is a bounded distributive lattice.
(2) Equivalence (1) holds for all formulas $\varphi, \psi \in \mathsf{MTL}(D, V)$.

Proposition 4 gives a precise characterization of when the identity (1) applies. This characterization is axiomatic and identifies the class of bounded distributive lattices as the most general class for which the identity is valid. One important implication is that monitors that are based on this identity cannot be used for other semirings such as $(\mathbb{R}, +, \cdot, 0, 1)$ and $(\mathbb{N}, +, \cdot, 0, 1)$.

**Example 5 (Uncertainty).** We want to identify a notion of quantitative truth values in situations where we interpret formulas over a signal $x[n]$ that is not known with perfect accuracy, but we can put an upper and lower bound on each sample, i.e., $a \leq x[n] \leq b$. For example, suppose that we know that $99.9 \leq x[0] \leq 100.1$ and we want to evaluate the atomic predicate $p =$ "$x \geq 99$" at time 0. The truth value can be taken to be the interval [0.9, 1.1] in this case, since there is uncertainty in the distance of signal value from the threshold.

More concretely, this situation of uncertain input signal can arise in the monitoring of systems where the raw signal is captured at one site, then compressed and transmitted to another site for monitoring. In many resource-constrained settings (e.g., certain IoT systems), the signal has to be compressed with a lossy compression scheme in order to meet network bandwidth constraints. So, at the monitoring site, the exact signal values are not known but can possibly be placed within intervals (depending on the used compression scheme).

In order to model this kind of uncertainty, we consider the set $\mathcal{I}(\mathbb{R}^{\pm\infty})$ of intervals of the form $[a, b]$ with $a \leq b$ and $a, b \in \mathbb{R}^{\pm\infty}$. An interval $[a, b] \subseteq \mathbb{R}^{\pm\infty}$ can be thought of as an uncertain truth value (it can be any one of those contained in $[a, b]$). For intervals $[a, b]$ and $[c, d]$ we define $[a, b] \oplus [c, d] = [\max(a, c), \max(b, d)]$ and $[a, b] \otimes [c, d] = [\min(a, c), \min(b, d)]$. An interval of the form $[a, a]$ is equal to the singleton set $\{a\}$. The structure $(\mathcal{I}(\mathbb{R}^{\pm\infty}), \oplus, \otimes, \{-\infty\}, \{\infty\})$ is a semiring.

The semiring $\mathcal{I}(\mathbb{R}^{\pm\infty})$ is a partial order (more specifically, it is a bounded distributive lattice) and therefore does not fit existing monitoring frameworks that consider only linear orders (e.g., the max-min semiring $\mathbb{R}^{\pm\infty}$ of the extended reals and the associated sliding-max/min algorithms).

### 3 Symbolic Quantitative Traces and Languages

In this section we start with our investigation of how to generalize the "robustness degree" of [22] to our abstract algebraic setting. The result of [22] that relates the robustness degree with the robust semantics is an inequality. For this reason, we focus on idempotent semirings, for which there is a natural partial order $\leq$ that is induced by semiring addition ($x \leq y$ iff $x + y = y$). Since our approach is abstract algebraic (i.e., axiomatic), we have no notion of real-valued distance between elements of $D$. Moreover, $V$ does not need to be a semiring of real numbers. Instead, we rely on the intuition that for an atomic predicate $p : D \to V$ and a data item $d \in D$, the value $p(d)$ gives a degree of truth or falsity. We propose using *symbolic traces* $\mathbf{x} = p_0 p_1 \ldots p_{n-1}$, which are sequences of atomic predicates, in order to compactly represent sets of *concrete traces*, which are sequences of data items. If each $p_i$ represents a subset $S_i \subseteq D$, then $\mathbf{x}$ represents the set $L = S_0 \times S_1 \times \cdots \times S_{n-1} = \{v_0 v_1 \ldots v_{n-1} \mid v_i \in S_i \text{ for each } i\}$ of concrete traces. Moreover, given a concrete trace $u = u_0 u_1 \ldots u_{n-1} \in D^n$, we can use the value $p_0(u_0) \cdot p_1(u_1) \cdots p_{n-1}(u_{n-1}) \in V$ as a quantitative measure of how close the trace $u$ is to the set of traces $L$. We propose the interpretation of a formula $\varphi$ as a *language of symbolic traces*. This will allow us to define the "closeness" of a trace $u \in D^n$ to the specification $\varphi$ as a (semiring) sum of all the closeness values w.r.t. each symbolic trace in the symbolic language of $\varphi$. We will also see that this interpretation of a formula $\varphi$ as a symbolic language is compatible with the standard interpretation of $\varphi$ as a set of concrete traces. Using these definitions we obtain a generalization of the theorem of [22] that relates the robustness degree with the robust semantics. Additionally, we characterize precisely the class of semirings for which this generalization is possible.

Let $V$ be an idempotent semiring. For predicates $p, q : D \to V$ we define $p \leq q$ if $p(d) \leq q(d)$ for every $d \in D$. The intuition for $p \leq q$ is that $p$ is a stronger predicate than $q$. We write $\mathsf{F}(D, V)$ to denote the set of atomic quantitative predicates, which always includes the predicates $\mathbb{1}$ and $\mathbb{0}$. For symbolic traces $\mathbf{x}, \mathbf{y} \in \mathsf{F}(D, V)^\infty$ with $\lambda = |\mathbf{x}| = |\mathbf{y}|$ we define $\mathbf{x} \leq \mathbf{y}$ if $\mathbf{x}(i) \leq \mathbf{y}(i)$ for every $i < \lambda$. These relations $\leq$ on predicates and traces are partial orders. We define the *symbolic satisfaction relation* $\models$, where $\mathbf{x}, i \models \varphi$ says that the formula $\varphi : \mathsf{MTL}(D, V)$ is satisfied by the symbolic trace $\mathbf{x} \in \mathsf{F}(D, V)^\infty$ at position $i < |\mathbf{x}|$. For atomic formulas, we put $\mathbf{x}, i \models p$ iff $\mathbf{x}(i) \leq p$. The definition is given by induction on $\varphi$ in the usual way. For a formula $\varphi : \mathsf{MTL}(D, V)$, length $\lambda \in \omega \cup \{\omega\}$ and a position $i < \lambda$, we define the *symbolic language* $\mathsf{SL}(\varphi, \lambda, i) = \{\mathbf{x} \in \mathsf{F}(D, V)^\lambda \mid \mathbf{x}, i \models \varphi\}$. For nonempty finite traces $\mathbf{x} \in \mathsf{F}(D, V)^n$ and $u \in D^n$ of the same length, we define $\mathbf{x}[u] = \prod_{i=1}^n \mathbf{x}(i)(u(i))$, where $n = |\mathbf{x}| = |u|$. Since the semiring multiplication is monotone w.r.t. $\leq$, we see that $\mathbf{x} \leq \mathbf{y}$ implies $\mathbf{x}[u] \leq \mathbf{y}[u]$ for every $u \in D^n$. Informally, the value $\mathbf{x}[u]$ quantifies how close the concrete trace $u$ is to the symbolic trace $\mathbf{x}$.

**Example 6.** Let $D = \mathbb{R}$ and $V = \mathbb{R}^{\pm\infty}$. For $c \in \mathbb{R}$, the predicate $p =$ "$x \geq c$" is defined by $p(d) = d - c$ for every $d \in D$. The predicate $q =$ "$x \leq c$" is given by $q(d) = c - d$ for every $d \in D$. For the symbolic trace $\mathbf{x} =$ "$x \geq 1$" "$x \leq 5$" "$x \geq 2$" and the concrete trace $u = 3\,6\,8$ we get that $\mathbf{x}[u] = \min(2, -1, 6) = -1$.

Let $c, d \in \mathbb{R}$. For the predicates $p =$ "$x \geq c$" and $q =$ "$x \geq d$" we have that $p \leq q$ iff $d \leq c$. Similarly, for the predicates $p =$ "$x \leq c$" and $q =$ "$x \leq d$" it holds that $p \leq q$ iff $c \leq d$. Finally, notice that the predicates "$x \geq c$" and "$x \leq d$" are incomparable. Consider $\mathbf{y} =$ "$x \geq 0$" "$x \leq 7$" "$x \geq 1$" and observe that $\mathbf{x} \leq \mathbf{y}$.

For the formula $\varphi = p \wedge \mathsf{F}_1 q$, where $p$ and $q$ are atomic predicates, we have that $\mathsf{SL}(\varphi, 2, 0) = \{p'q' \in \mathsf{F}(D, V)^2 \mid p' \leq p \text{ and } q' \leq q\}$.

The definition of the robustness degree in [22] involves the value $-\mathsf{dist}(u, L) = -\inf_{v \in L} \mathsf{dist}(u, v) = \sup_{v \in L} -\mathsf{dist}(u, v)$, where $u$ is a trace, $L$ is a set of traces, and $\mathsf{dist}$ is a metric. Notice that this is a supremum over a potentially infinite set. The semirings that we have considered so far have an addition operation that can model a *finitary* supremum. In order to model an *infinitary* supremum, we need to consider semirings that have an infinitary addition operation. A *complete semiring* is an algebraic structure $(V, +, \sum, \cdot, 0, 1)$, where $\sum_{i \in I} x_i$ is the sum of the $I$-indexed tuple of elements $(x_i)_{i \in I}$, that satisfies: (1) $\sum_{i \in \emptyset} x_i = 0$, $\sum_{i \in \{j\}} x_i = x_j$, $\sum_{i \in \{j,k\}} x_i = x_j + x_k$ for $j \neq k$, and $\sum_{k \in K} \sum_{i \in I_k} x_i = \sum_{i \in I} x_i$ where $I = \bigcup_{k \in K} I_k$ and the index sets $(I_k)_{k \in K}$ are pairwise disjoint, (2) $(V, \cdot, 1)$ is a monoid, (3) the infinite distributivity properties $(\sum_{i \in I} x_i) \cdot y = \sum_{i \in I} (x_i y)$ and $x \cdot (\sum_{i \in I} y_i) = \sum_{i \in I} (x y_i)$ hold for every index set $I$ and all $x_i, y \in V$, and (4) 0 is an annihilator for multiplication. A complete semiring $V$ is *idempotent* if $\sum_{i \in I} x_i = x$ for every non-empty index set $I$ with $x_i = x$ for every $i \in I$. For example, $(\mathbb{R}^{\pm\infty}, \max, \sup, \min, -\infty, +\infty)$ is an idempotent complete semiring. For a formula $\varphi : \mathsf{MTL}(D, V)$, a trace $u \in D^+$ and $i < n = |u|$, we define

$$\mathsf{val}(\varphi, u, i) = \textstyle\sum_{\mathbf{x} \in \mathsf{SL}(\varphi, n, i)} \mathbf{x}[u]. \tag{2}$$

Informally, $\mathsf{val}(\varphi, u, i)$ is a measure of how close the trace $u$ is to satisfying $\varphi$ at position $i$. It is an abstract algebraic variant of the robustness degree [22].

**Theorem 7 (Approximation).** Let $D$ be a set of data items and $V$ be an idempotent complete semiring. Then, the following are equivalent:
(1) The multiplication of $V$ is idempotent and 1 is the top element of $V$.

(2) For every $\varphi : \mathsf{MTL}(D, V)$, $u \in D^+$ and $i < |u|$, $\mathsf{val}(\varphi, u, i) \le \rho(\varphi, u, i)$.

*Proof.* Assume that (1) holds. Let $n \ge 1$ be an integer. For a symbolic language $\mathcal{L} \subseteq \mathsf{F}(D, V)^n$ and for $u \in D^n$, we define $\mathsf{val}(\mathcal{L}, u) = \sum_{\mathbf{x} \in \mathcal{L}} \mathbf{x}(u)$. Let $\{\mathcal{L}_i\}_{i \in I}$ be a collection of languages with $\mathcal{L}_i \subseteq \mathsf{F}(D, V)^n$. Then,

$$\mathsf{val}(\textstyle\bigcup_{i \in I} \mathcal{L}_i, u) = \sum_{\mathbf{x} \in \bigcup_{i \in I} \mathcal{L}_i} \mathbf{x}[u] \le \sum_{i \in I} \sum_{\mathbf{x} \in \mathcal{L}_i} \mathbf{x}[u] = \sum_{i \in I} \mathsf{val}(\mathcal{L}_i, u). \quad (3)$$

For symbolic languages $\mathcal{L}_1, \mathcal{L}_2 \subseteq \mathsf{F}(D, V)^n$, define $\mathcal{L} = \mathcal{L}_1 \cap \mathcal{L}_2$, $\mathcal{L}_1' = \mathcal{L}_1 \setminus \mathcal{L}_2$ and $\mathcal{L}_2' = \mathcal{L}_2 \setminus \mathcal{L}_1$. Then, $\mathcal{L}_1 = \mathcal{L}_1' \cup \mathcal{L}$ and $\mathcal{L}_2 = \mathcal{L}_2' \cup \mathcal{L}$. The languages $\mathcal{L}_1', \mathcal{L}_2', \mathcal{L}$ are pairwise disjoint. So, we have that $\mathsf{val}(\mathcal{L}_1, u) = x + z$ and $\mathsf{val}(\mathcal{L}_2, u) = y + z$, where $x = \mathsf{val}(\mathcal{L}_1', u)$, $y = \mathsf{val}(\mathcal{L}_2', u)$ and $z = \mathsf{val}(\mathcal{L}, u)$. It follows that

$$\mathsf{val}(\mathcal{L}_1 \cap \mathcal{L}_2, u) = z = zz \le (x + z)(y + z) = \mathsf{val}(\mathcal{L}_1, u) \cdot \mathsf{val}(\mathcal{L}_2, u) \quad (4)$$

by the idempotence of multiplication. This property extends to $\mathsf{val}(\mathcal{L}_1 \cap \cdots \cap \mathcal{L}_k, u) \le \mathsf{val}(\mathcal{L}_1, u) \cdots \mathsf{val}(\mathcal{L}_k, u)$. Now, we will prove (2) by induction on $\varphi$.

- For the base case we have $\mathsf{SL}(p, n, i) = \{\mathbf{x} \in \mathsf{F}(D, V)^n \mid \mathbf{x}(i) \le p\}$. Define $\mathbf{y} \in \mathsf{F}(D, V)^n$ by $\mathbf{y}(i) = p$ and $\mathbf{y}(j) = \mathbb{1}$ for every $j \ne i$. That is, $\mathbf{y} = \mathbb{1}^i \, p \, \mathbb{1}^{n-i-1}$. For every $\mathbf{x} \in \mathsf{SL}(p, n, i)$ we have $\mathbf{x}(i) \le p$ and therefore $\mathbf{x} \le \mathbf{y}$ (since 1 is the top element of $V$). It follows that $\mathbf{x}[u] \le \mathbf{y}[u] = p(u(i))$. So, $\mathsf{val}(p, u, i) = \sum_{\mathbf{x} \in \mathsf{SL}(p,n,i)} \mathbf{x}[u] \le p(u(i)) = \rho(p, u, i)$.
- For the case of disjunction, we have $\mathsf{SL}(\varphi \vee \psi, n, i) = \mathsf{SL}(\varphi, n, i) \cup \mathsf{SL}(\psi, n, i)$. It follows that $\mathsf{val}(\varphi \vee \psi, u, i) \le \mathsf{val}(\varphi, u, i) + \mathsf{val}(\psi, u, i) \le \rho(\varphi, u, i) + \rho(\psi, u, i) = \rho(\varphi \vee \psi, u, i)$ by the induction hypothesis and (3).
- For the case of conjunction we observe that $\mathsf{val}(\varphi \wedge \psi, u, i) = \mathsf{val}(\mathsf{SL}(\varphi, n, i) \cap \mathsf{SL}(\psi, n, i), u) \le \mathsf{val}(\varphi, u, i) \cdot \mathsf{val}(\psi, u, i) \le \rho(\varphi, u, i) \cdot \rho(\psi, u, i) = \rho(\varphi \wedge \psi, u, i)$ by the induction hypothesis and (4).

The rest of the cases $\mathsf{S}$, $\bar{\mathsf{S}}$, $\mathsf{U}$, $\bar{\mathsf{U}}$ can be dealt with similarly using (3) and (4). The proof that (2) implies (1) is not too difficult, and we therefore omit it. □

Theorem 7 could be considered an abstract algebraic counterpart of the result of [22] (page 4268, Theorem 13) for discrete finite traces. We will discuss later how it can be used to obtain the original result (for the max-min semiring $\mathbb{R}^{\pm\infty}$) as a corollary. Additionally, Theorem 7 gives a precise equational characterization of the class of semirings for which the relationship between the two semantics holds.

Let $D$ be a set of data items, $V$ be a semiring and $h : V \to \mathbb{B}$. For a formula $\varphi : \mathsf{MTL}(D, V)$, length $\lambda \in \omega \cup \{\omega\}$ and $i < \lambda$, we define the *concrete trace language* $\mathsf{CL}_h(\varphi, \lambda, i) = \{u \in D^\lambda \mid u, i \models_h \varphi\}$. For a symbolic trace $\mathbf{x} \in \mathsf{F}(D, V)^\lambda$, we define its (concrete) *trace language* by $\mathsf{CL}_h(\mathbf{x}) = \{u \in D^\lambda \mid u \models_h \mathbf{x}\}$, where $u \models_h \mathbf{x}$ means that $u(i) \models_h \mathbf{x}(i)$ for every $i < n$. Lemma 8 below establishes a correspondence between the symbolic and concrete language of a formula $\varphi$, which we need to connect Theorem 7 to the concrete setting of [22].

**Lemma 8 (Concrete and Symbolic Languages).** Let $D$ be a set of data items, $V$ be an idempotent semiring with top element 1, and $h : V \to \mathbb{B}$ be a semiring homomorphism. For every formula $\varphi : \mathsf{MTL}(D, V)$, length $\lambda \in \omega \cup \{\omega\}$, and position $i < \lambda$, it holds that $\mathsf{CL}_h(\varphi, \lambda, i) = \bigcup_{\mathbf{x} \in \mathsf{SL}(\varphi, \lambda, i)} \mathsf{CL}_h(\mathbf{x})$.

## 4 Relationship with robust semantics

In this section, we consider the concrete quantitative setting where $V$ is the max-min semiring $\mathbb{R}^{\pm\infty}$. We will obtain the result of [22] that relates the robustness degree with the robust semantics as a consequence of Theorem 7.

A *metric space* is a set $M$ together with a function $\mathsf{dist} : M \times M \to \mathbb{R}_{\geq 0}$, called *metric*, satisfying: (1) $\mathsf{dist}(x, y) = 0$ iff $x = y$ for all $x, y \in M$, (2) $\mathsf{dist}(x, y) = \mathsf{dist}(y, x)$ for all $x, y \in M$, and (3) $\mathsf{dist}(x, z) \leq \mathsf{dist}(x, y) + \mathsf{dist}(y, z)$ for all $x, y, z \in M$. Given a metric $\mathsf{dist}$ on $M$ we define the *distance function* $\mathsf{Dist}$ as follows:

$$\mathsf{dist} : M \times \mathcal{P}(M) \to \mathbb{R}_{\geq 0}^{\infty} \qquad \mathsf{Dist} : M \times \mathcal{P}(M) \to \mathbb{R}^{\pm\infty}$$

$$\mathsf{dist}(x, S) = \inf\nolimits_{y \in S} \mathsf{dist}(x, y) \qquad \mathsf{dist}(x, \emptyset) = \infty \qquad \mathsf{Dist}(d, S) = \begin{cases} -\mathsf{dist}(d, S), & \text{if } d \notin S \\ \mathsf{dist}(d, {\sim}S), & \text{if } d \in S \end{cases}$$

where ${\sim}S = M \setminus S$ is the complement of $S$. Notice that $\mathsf{Dist}(x, \emptyset) = -\infty$.

Let $D$ be a metric space of points (data items). Let $\mathsf{p}$ be a propositional letter (symbol), and $\mathcal{O}(\mathsf{p}) \subseteq D$ be its interpretation, that is, the set of points for which $\mathsf{p}$ is true. The corresponding quantitative predicate is $p : D \to \mathbb{R}^{\pm\infty}$ given by $p(d) = \mathsf{Dist}(d, \mathcal{O}(\mathsf{p}))$ for every $d \in D$. Given the metric $\mathsf{dist}$ on $D$, we obtain a metric $\mathsf{dist} : D^\lambda \times D^\lambda \to \mathbb{R}_{\geq 0}^{\infty}$ (on the set of traces of length $\lambda$, where $\lambda \in \omega \cup \{\omega\}$) as follows: $\mathsf{dist}(u, v) = \sup_{i<\lambda} \mathsf{dist}(u(i), v(i))$. Let $\mathsf{CL}_{\mathcal{O}}(\varphi, n, i) = \{u \in D^n \mid u, i \models_{\mathcal{O}} \varphi\}$ be the set of traces (of length $n$) that satisfy $\varphi$ at $i$ (defined using the interpretation function $\mathcal{O}$). Corollary 9 below was proved in [22]. We will give a proof that relies on the algebraic variant that we presented earlier.

**Corollary 9.** Let $D$ be a set of data items, and $V = \mathbb{R}^{\pm\infty}$. Let $\varphi : \mathsf{MTL}(D, V)$, $u \in D^n$ and $i < n$ (where $n \geq 1$). Then, $-\mathsf{dist}(u, \mathsf{CL}_{\mathcal{O}}(\varphi, n, i)) \leq \rho(\varphi, u, i)$.

*Proof.* We will use the semiring $\mathbb{R}_{\pm 0}^{\pm\infty} \cong \mathbb{B} \times \mathbb{R}_{\geq 0}^{\infty}$ instead of $\mathbb{R}^{\pm\infty}$, so that the value 0 is not ambiguous (it can be either true or false when we use $\mathbb{R}^{\pm\infty}$). That is, we will have a positive zero $+0$ (true) and a negative zero $-0$ (false). The semiring homomorphism $h : \mathbb{R}_{\pm 0}^{\pm\infty} \to \mathbb{B}$ sends the positive (resp., negative) elements to $\top$ (resp., $\bot$). We will interpret a predicate symbol $\mathsf{p}$ as the quantitative predicate $p : D \to \mathbb{R}_{\pm 0}^{\pm\infty}$ given by $p(d) = -\mathsf{dist}(d, \mathcal{O}(\mathsf{p}))$ if $d \notin \mathcal{O}(\mathsf{p})$ and $p(d) = +\mathsf{dist}(d, {\sim}\mathcal{O}(p))$ if $d \in \mathcal{O}(\mathsf{p})$. Using these definitions, the satisfaction relations $\models_{\mathcal{O}}$ and $\models_h$ are the same, hence $\mathsf{CL}_{\mathcal{O}}$ and $\mathsf{CL}_h$ are the same. Now,

$$\begin{aligned}
\mathsf{dist}(u, \mathsf{CL}_h(\varphi, n, i)) &= \mathsf{dist}(u, \textstyle\bigcup_{\mathbf{x} \in \mathsf{SL}(\varphi, n, i)} \mathsf{CL}_h(\mathbf{x})) && \text{[Lemma 8]} \\
&= \inf_{\mathbf{x} \in \mathsf{SL}(\varphi, n, i)} \inf_{v \in \mathsf{CL}_h(\mathbf{x})} \sup_{i<n} \mathsf{dist}(u(i), v(i)) && \text{[def. of dist]} \\
&\geq \inf_{\mathbf{x} \in \mathsf{SL}(\varphi, n, i)} \sup_{i<n} \inf_{v \in \mathsf{CL}_h(\mathbf{x})} \mathsf{dist}(u(i), v(i)) && [\sup\inf \leq \inf\sup] \\
&= \inf_{\mathbf{x} \in \mathsf{SL}(\varphi, n, i)} \sup_{i<n} \inf_{v(i) \in \mathcal{O}(\mathbf{x}(i))} \mathsf{dist}(u(i), v(i)) && \text{[def. of CL]} \\
&= \inf_{\mathbf{x} \in \mathsf{SL}(\varphi, n, i)} \sup_{i<n} \mathsf{dist}(u(i), \mathcal{O}(\mathbf{x}(i))). && \text{[def. of dist]}
\end{aligned}$$

By negating the above inequality we get that

$$-\mathsf{dist}(u, \mathsf{CL}_h(\varphi, n, i)) \le \sup_{\mathbf{x} \in \mathsf{SL}(\varphi,n,i)} \inf_{i<n} - \mathsf{dist}(u(i), \mathcal{O}(\mathbf{x}(i))),$$

which is $\le \sum_{\mathbf{x} \in \mathsf{SL}(\varphi,n,i)} \mathbf{x}[u] = \mathsf{val}(\varphi, u, i)$. From Theorem 7 we get $\mathsf{val}(\varphi, u, i) \le \rho(\varphi, u, i)$ and therefore $-\mathsf{dist}(u, \mathsf{CL}_{\mathcal{O}}(\varphi, n, i)) \le \rho(\varphi, u, i)$. □

From Corollary 9 we can also obtain $\rho(\varphi, u, i) \le \mathsf{dist}(u, {\sim}\mathsf{CL}_{\mathcal{O}}(\varphi, n, i))$. This inequality is equivalent to $-\mathsf{dist}(u, {\sim}\mathsf{CL}_{\mathcal{O}}(\varphi, n, i)) \le -\rho(\varphi, u, i)$, which in turn is equivalent to $-\mathsf{dist}(u, \mathsf{CL}_{\mathcal{O}}({\sim}\varphi, n, i)) \le \rho({\sim}\varphi, u, i)$. The operation $\sim$ on formulas is a pseudo-negation, that is, ${\sim}\varphi$ is the formula that results by "dualizing" all connectives and negating the atomic predicates. This operation is meaningful for the semiring $\mathbb{R}^{\pm\infty}$. The final inequality is an instance of Corollary 9 for ${\sim}\varphi$.

Theorem 7 and Corollary 9 are not used later for the monitoring algorithm. The significance of our theorem is that it can be instantiated to give the existing result from [22]. This serves as a sanity check for our algebraic framework and it supports the semiring-based semantics of Sect. 2.

### 5 Online Monitoring

For an infinite input trace $u \in D^\omega$, the output of the monitor for the time instant $t$ should be $\rho(\varphi, u, t)$, but the monitor has to compute it by observing only a finite prefix of $u$. In order for the output value of the monitor to agree with the standard temporal semantics over infinite traces we may need to delay an output item until some part of the future input is seen. For example, in the case of $\mathsf{F}_1 p$ we need to wait for one time unit: the output at time $t$ is given after the input item at time $t+1$ is seen. In other words, the monitor for $\mathsf{F}_1 p$ has a *delay* (the output is falling behind the input) of one time unit. Symmetrically, we can allow monitors to emit output early when the correct value is known. For example, the output value for $\mathsf{P}_1 p$ is 0 in the beginning and the value at time $t$ is already known from time $t-1$. So, we also allow monitors to have negative delay (the output is running ahead of the input). The function $\mathsf{dl} : \mathsf{MTL} \to \mathbb{Z}$ gives the amount of delay required to monitor a formula. It is defined by $\mathsf{dl}(p) = 0$ and

$$\mathsf{dl}(\varphi \wedge \psi) = \max(\mathsf{dl}(\varphi), \mathsf{dl}(\psi)) \qquad \mathsf{dl}(\varphi\, \mathsf{S}_{[a,b]}\, \psi) = \max(\mathsf{dl}(\varphi), \mathsf{dl}(\psi)) - a$$
$$\mathsf{dl}(\varphi\, \mathsf{S}_{[a,\infty)}\, \psi) = \max(\mathsf{dl}(\varphi), \mathsf{dl}(\psi)) - a \quad \mathsf{dl}(\varphi\, \mathsf{U}_{[a,b]}\, \psi) = \max(\mathsf{dl}(\varphi), \mathsf{dl}(\psi)) + b.$$

The monitor $\mathtt{TL}(\varphi)$ for a formula $\varphi$ is a variant of a Mealy machine. If $\mathsf{dl}(\varphi) = 0$, the $\mathtt{TL}(\varphi)$ is precisely a Mealy machine (one output item per input item) with inputs $D$ and outputs $V$. If $\ell = \mathsf{dl}(\varphi) > 0$, then $\mathtt{TL}(\varphi)$ emits no output for the first $\ell$ steps and then behaves like a Mealy machine. If $\ell = \mathsf{dl}(\varphi) < 0$, then $\mathtt{TL}(\varphi)$ emits $\ell$ items upon initialization and continues to behave like a Mealy machine.

Let $A$ and $B$ be sets. A *monitor* of type $\mathsf{M}(A, B)$ is a state machine $G = (\mathsf{St}, \mathsf{init}, \mathsf{o}, \mathsf{next}, \mathsf{out})$, where $\mathsf{St}$ is a set of *states*, $\mathsf{init} \in \mathsf{St}$ is the *initial state*, $\mathsf{o} \in B^*$ is the *initial output*, $\mathsf{next} : \mathsf{St} \times A \to \mathsf{St}$ is the *transition function*, and $\mathsf{out} : \mathsf{St} \times A \to \mathsf{Opt}(B)$ is the *output function*, where $\mathsf{Opt}(B) = B \cup \{\mathsf{nil}\}$.

$$\begin{array}{lll}
\texttt{map}(op) : \mathsf{M}(A,B) & \texttt{aggr}(b, op) : \mathsf{M}(A,B) & \texttt{emit}(n,v) : \mathsf{M}(A,A) \\
\mathsf{St} = \texttt{Unit} & \mathsf{St} = B & \mathsf{St} = \texttt{Unit} \\
\mathsf{init} = \mathtt{u} & \mathsf{init} = b & \mathsf{init} = \mathtt{u} \\
\mathsf{o} = \varepsilon & \mathsf{o} = \varepsilon & \mathsf{o} = v^n \\
\mathsf{next}(s,a) = s & \mathsf{next}(s,a) = op(s,a) & \mathsf{next}(s,a) = s \\
\mathsf{out}(s,a) = op(a) & \mathsf{out}(s,a) = op(s,a) & \mathsf{out}(s,a) = a
\end{array}$$

$$\begin{array}{lll}
\texttt{ignore}(n) : \mathsf{M}(A,A) & \texttt{wnd}(n, v, op) : \mathsf{M}(A,A) & \texttt{wndV}(n, op) : \mathsf{M}(A,A) \\
\mathsf{St} = [0,n] & \mathsf{St} = \mathsf{Buf}(A) & \mathsf{St} = \mathsf{Buf}(A) \\
\mathsf{init} = 0 & \mathsf{init} = \mathsf{Buf}(n,v) & \mathsf{init} = \mathsf{Buf}() \\
\mathsf{o} = \varepsilon & \mathsf{o} = \varepsilon & \mathsf{o} = \varepsilon \\
\mathsf{next}(s,a) = s+1, \text{ if } s < n & \mathsf{next}(s,a) = s.\mathsf{ins}(a) & \mathsf{next}(s,a) = s.\mathsf{ins}(a) \\
\mathsf{next}(s,a) = s, \text{ if } s = n & \mathsf{out}(s,a) = s.\mathsf{ins}(a).\mathsf{agg}(op) & \mathsf{out}(s,a) = \varepsilon, \text{ if } \mathsf{size}(s) < n-1 \\
\mathsf{out}(s,a) = \mathsf{nil}, \text{ if } s < n & & \mathsf{out}(s,a) = s.\mathsf{ins}(a).\mathsf{agg}(op), \text{ o/w} \\
\mathsf{out}(s,a) = a, \text{ if } s = n & &
\end{array}$$

Fig. 3: Basic building blocks for constructing temporal quantitative monitors.

In Fig. 3 we give several examples of simple monitors that can be used as building blocks. The monitor $\texttt{map}(op)$ applies the function $op : A \to B$ elementwise. The monitor $\texttt{aggr}(b, op)$ applies a running aggregation to the input trace that is specified by the initial aggregate $b : B$ and the aggregation function $op : B \times A \to B$ (similar to the fold combinator used in functional programming). The monitor $\texttt{emit}(n, v)$ emits $n$ copies of the value $v \in A$ upon initialization and then echoes the input trace. The monitor $\texttt{ignore}(n)$ discards the first $n$ items of the trace and proceeds to echo the rest of the trace. The monitor $\texttt{wnd}(n, v, op)$ performs an aggregation, given by the associative function $op : A \times A \to A$, over a sliding window of size $n$. It initializes the window using the value $v : A$ and emits output at the arrival of every item. The monitor $\texttt{wndV}(n, op)$ is different in that it starts with an empty window and it only starts emitting output when the window fills up with $n$ items. We will combine monitors using the operations *serial composition* $\gg$ and *parallel composition* $\texttt{par}$. In the serial composition $G \gg H$ the output trace of $G$ is propagated as input trace to $H$. In the parallel composition $\texttt{par}(G, H)$ the input trace to copied to two concurrently executing monitors $G$ and $H$ and their output traces are combined. Both combinators $\gg$ and $\texttt{par}$ are given by variants of the product construction on state machines. In the case of $\texttt{par}$ the output traces of $G$ and $H$ may not be synchronized (one may be ahead of the other), which requires some bounded buffering in order to properly align them. The construction for $\texttt{par}$ is described in [37]. Some variants of the combinators of Figure 3 are part of the StreamQL language [29], which has been proposed for the processing of streaming time series.

The identities of Fig. 2 suggest that MTL monitoring can be reduced to a small set of computational primitives. In fact, the primitives described earlier are sufficient to specify the monitors, as shown in Fig. 4. We write $\pi_1 : A \times B \to A$ for the left projection and $\pi_2 : A \times B \to B$ for the right projection.

Let $u \in D^+$ and $n = |u|$. If $n > a$ then $\rho(\varphi \mathsf{S}_{[0,a]} \psi, u, n-1) = \rho(\varphi \mathsf{S} \psi, v, a)$, where $v$ is the suffix of $u$ with $a + 1$ items. If $n \leq a$ then $\rho(\varphi \mathsf{S}_{[0,a]} \psi, u, n-1) =$

$$
\begin{aligned}
\texttt{TL}(p) &= \texttt{map}(p) \\
\texttt{TL}(\varphi \vee \psi) &= \texttt{par}(\texttt{TL}(\varphi), \texttt{TL}(\psi)) \gg \texttt{map}(+) \\
\texttt{TL}(\mathsf{P}\varphi) &= \texttt{TL}(\varphi) \gg \texttt{aggr}(0, +) \\
\texttt{TL}(\mathsf{P}_a\varphi) &= \texttt{TL}(\varphi) \gg \texttt{emit}(a, 0) \\
\texttt{TL}(\mathsf{P}_{[a,\infty)}\varphi) &= \texttt{TL}(\mathsf{P}_a\mathsf{P}\varphi) \\
\texttt{TL}(\varphi \,\mathsf{S}\, \psi) &= \texttt{par}(\texttt{TL}(\varphi), \texttt{TL}(\psi)) \gg \texttt{aggr}(0, opS) \\
opS &: V \times (V \times V) \to V, \text{ where} \\
opS(s, \langle x, y\rangle) &= (s \cdot x) + y \\
\texttt{TL}(\varphi \,\mathsf{S}_{[a,\infty)}\, \psi) &= \texttt{TL}(\mathsf{P}_a(\varphi \,\mathsf{S}\, \psi) \wedge \mathsf{H}_{[0,a-1]}\varphi) \\
\texttt{TL}(\varphi \,\mathsf{S}_{[0,b]}\, \psi) &= \texttt{par}(\texttt{TL}(\varphi), \texttt{TL}(\psi)) \gg \\
&\qquad \texttt{wnd}(b+1, 0, \otimes_{\mathsf{S}}) \gg \texttt{map}(\pi_2) \\
\texttt{TL}(\varphi \,\mathsf{S}_{[a,b]}\, \psi) &= \texttt{TL}(\mathsf{P}_a(\varphi \,\mathsf{S}_{[0,b-a]}\, \psi) \wedge \mathsf{H}_{[0,a-1]}\varphi) \\
\texttt{TL}(\mathsf{F}_a\varphi) &= \texttt{TL}(\varphi) \gg \texttt{ignore}(a) \\
\texttt{TL}(\mathsf{F}_{[a,b]}\varphi) &= \texttt{TL}(\mathsf{F}_b\mathsf{P}_{[0,b-a]}\varphi) \\
\texttt{TL}(\varphi \,\mathsf{U}_{[0,b]}\, \psi) &= \texttt{par}(\texttt{TL}(\varphi), \texttt{TL}(\psi)) \gg \\
&\qquad \texttt{wndV}(b+1, \otimes_{\mathsf{U}}) \gg \texttt{map}(\pi_2) \\
\texttt{TL}(\varphi \,\mathsf{U}_{[a,b]}\, \psi) &= \texttt{TL}(\mathsf{G}_{[0,a-1]}\varphi \wedge \mathsf{F}_a(\varphi \,\mathsf{U}_{[0,b-a]}\, \psi))
\end{aligned}
$$

```
// fill buffer with v (initial values)
T[n] buf ← [n; v]
// calculate partial aggregates
for i ← n − 2 to 0 do
  | buf[i] ← buf[i] ⊗ buf[i + 1]
// initial total aggregate
T agg ← buf[0]
Nat m ← 0 // size of new block
T z ← nil // aggregate of new block
Function Add(T d):
    if m = n then // full new block
        // convert new block to old
        for i ← n − 2 to 1 do
          | buf[i] ← buf[i] ⊗ buf[i + 1]
        m ← 0 // empty new block
        z ← nil
    // evict oldest item, replace with d
    buf[m] ← d
    m ← m + 1 // new block enlarged
    z ← z ⊗ d // where nil ⊗ d = d
    if m < n then
      | agg ← buf[m] ⊗ z
    else // m = n
      | agg ← z
```

Fig. 4: Online monitors for bounded-future MTL formulas & sliding aggregation.

$\rho(\varphi \,\mathsf{S}\, \psi, 0^{a+1-n}u, a)$. So, we can implement a monitor for the connective $\mathsf{S}_{[0,a]}$ by computing $\mathsf{S}$ over a window of exactly $a+1$ data items.

**Proposition 10 (Aggregation for $\mathsf{S}$, $\mathsf{U}$).** Let $V$ be a semiring. For every trace $u = u_0 u_1 \ldots u_{n-1} \in (V \times V)^+$ of length $n = |u|$, the values $\rho(\pi_1 \mathsf{S} \pi_2, u, n-1)$ and $\rho(\pi_1 \mathsf{U} \pi_2, u, 0)$ can be written as aggregates of the form $\pi_2(u_0 \otimes u_1 \otimes \cdots \otimes u_{n-1})$.

Proposition 10 justifies the translation of $\mathsf{S}_{[0,b]}/\mathsf{U}_{[0,b]}$ into monitors (Fig. 4). Now, we will describe the data structure that performs the sliding aggregation. It is used in Fig. 3 in the monitors `wnd` and `wndV`. The implementation is shown in Fig. 4. Suppose that the current window (of size $n$) is $[x_0, x_1, \ldots, x_{n-1}]$. We maintain a buffer of the form $[x_{n-m}, \ldots, x_{n-1}, y_0, \ldots, y_{n-1-m}]$, where the part $[x_{n-m}, \ldots, x_{n-1}]$ is the block of newer elements ("new block") and the part $[y_0, \ldots, y_{n-1-m}]$ contains aggregates of the older elements ("old block"). They satisfy the invariant $y_i = x_i \otimes \cdots \otimes x_{n-1-m}$ for every $i = 0, \ldots, n-1-m$. We also maintain the aggregate $z = x_{n-m} \otimes \cdots \otimes x_{n-1}$ of the new block. So, the overall aggregate of the window is $agg = y_0 \otimes z$. When a new item $d$ arrives, we evict the aggregate $y_0$ corresponding to the oldest item $x_0$ and replace it by $d$. Thus, the new block is expanded with the additional item $d$ and therefore we also update the aggregates $z$ and $agg$. When the new block becomes full (i.e., $m = n$) then we convert it to an old block by performing all partial aggregations from right to left. This conversion requires $n-1$ applications of $\otimes$, but it is performed once every $n$ items. So, the algorithm needs $O(1)$ amortized time-per-item.

**Theorem 11.** Let $D$ be a set of data items, $V$ be a semiring, and $\varphi : \mathsf{MTL}(D, V)$ be a bounded-future formula. The monitor $\texttt{TL}(\varphi) : \mathsf{M}(D, V)$ is a streaming algorithm that needs $O(2^{|\varphi|})$ space and $O(|\varphi|)$ amortized time-per-item.

*Proof.* The algorithm needs space that is exponential in the size of $\varphi$ because of the connectives of the form $X_{[a,\infty)}$ and $X_{[a,b]}$. The monitor uses buffers of size $a$ or $b-a$. Since the constants $a, b$ are written in binary notation, we need space that is exponential in the size. The $O(|\varphi|)$ amortized time per element hinges on the algorithm of Fig. 4, which is used for $\mathsf{S}_{[0,b]}$ and $\mathsf{U}_{[0,b]}$. As discussed earlier, this algorithm needs $O(1)$ amortized time-per-item.

### 6 Experimental Evaluation

We have implemented our semiring-based monitoring framework in Rust. We compare our implementation with the verified lattice-based monitors of [13] and the monitoring tool Reelay [40]. We perform our experiments using the $(\mathbb{R}^{\pm\infty}, \max, \min)$ semiring for truth values, which are approximately represented using 64-bit floating-point numbers.

We have observed that all three tools process items at a roughly constant rate. We summarize the performance of a monitor with the average time it takes to process one data item (i.e., amortized time-per-item). In Fig. 5, we consider formulas $X_{[0,n]}$, $X_n$, $X_{[n,2n]}$, $X_{[n,\infty)}$ where $X \in \{\mathsf{S}, \mathsf{P}\}$. We show the time-per-item for the monitors for $n = 1, 10, 10^2, 10^3, 10^4, 10^5, 10^6$. We have also evaluated how the monitors for future temporal connectives scale with respect to the constants in the intervals. In Fig. 6, we benchmark all tools using formulas from the Timescales benchmark [39]. Our monitors are generally more than 100 (resp., 10) times faster than Reelay (resp., the lattice-based tool of [13]).

The profiling tools Valgrind [38] and Heaptrack [41] are used to analyze the memory consumption of the monitors. Our Rust implementation, given a formula, begins by allocating a fixed amount of memory and does not allocate any more memory during the rest of the computation. Reelay allocates and de-allocates memory throughout its execution. The lattice-based monitor is implemented in OCaml (which is a garbage-collected language) and consumes a larger amount of memory. In Fig. 5, we plot the peak memory usage of the monitors. We note that our tool does not seem to be allocating an increasing amount of memory for $\mathsf{P}_n$ and similar formulas. This is because the corresponding monitor for $\mathsf{P}_n$ emits output as early as possible and therefore does not need to use a buffer. In the case of the lattice-based monitor and our tool, we observe that the memory consumption does not depend on the input trace (it only depends on the formula). In the case of Reelay, it appears that the memory consumption depends on the input trace. We have plotted the behavior for two different input traces: one that consists of an increasing sequence of values ("reelay-ascending"), and another one that is decreasing ("reelay-descending"). We have only measured the memory usage of Reelay for up to $n = 2^{13}$, as the execution becomes very slow beyond this value.

We use ***case studies*** from the automotive domain, which have been suggested as benchmarks for hybrid system verification [25]. The **A**utomatic Transmission System has two input signals (a throttle and a break) and three output signals: the gear sequence ($g_i$ for each gear $i$), the engine rotation speed (in

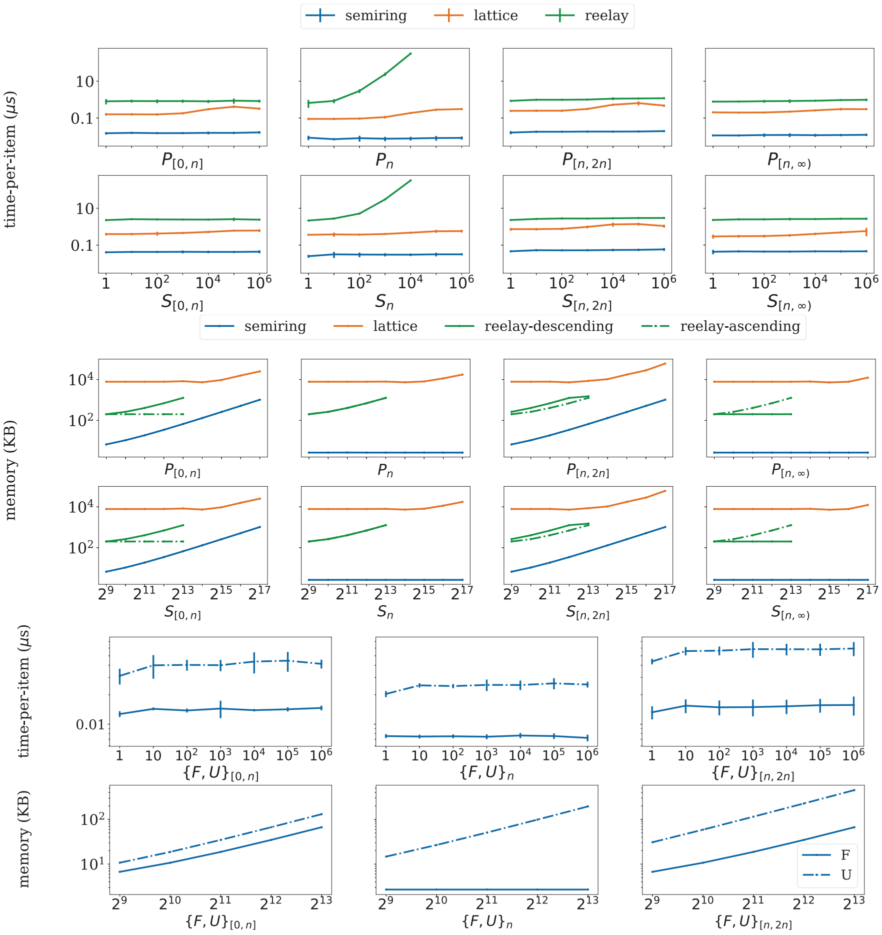

Fig. 5: Microbenchmark rpm, denoted $\omega$) and the vehicle speed (denoted $v$). Based on the suggestions in [25], we consider five properties: $A_1 = \omega < \overline{\omega}$, $A_2 = (\omega < \overline{\omega}) \wedge (v < \overline{v})$, $A_3 = g_1 \wedge \mathsf{Y}(g_2) \rightarrow \mathsf{YH}_{[0,2.5]} g_2$ (where $\mathsf{Y}$ is notation for $\mathsf{P}_1$), $A_4 = \mathsf{H}_{[0,5]}(\omega < \overline{\omega}) \rightarrow \mathsf{H}_{[0,2.5]}(v < \overline{v})$ and $A_5 = (v > \overline{v})\, \bar{\mathsf{S}}_{[0,1]}\, ((\omega > \overline{\omega})\, \bar{\mathsf{S}}_{[0,2]}\, ((\neg g_4)\, \bar{\mathsf{S}}_{[0,10]}\, ((\neg g_3)\, \bar{\mathsf{S}}\, ((\neg g_2)\, \bar{\mathsf{S}}\, (\neg g_1)))))$. All constants in the temporal connectives are in seconds, and we choose the constants $\overline{v} = 120$ and $\overline{\omega} = 4500$. Formula $A_3$ says that before changing from the second to the first gear, at least 2.5 seconds must first pass. Formula $A_4$ says that keeping the engine speed low enough should ensure that the vehicle does not exceed a certain speed. Formula $A_5$ says that changing the gear from the first to the fourth within 10 seconds, and then having the engine speed exceed $\overline{\omega}$ will cause the vehicle speed to exceed $\overline{v}$. The other case study is a **F**ault-Tolerant Fuel Control System. We monitor two properties. The first is

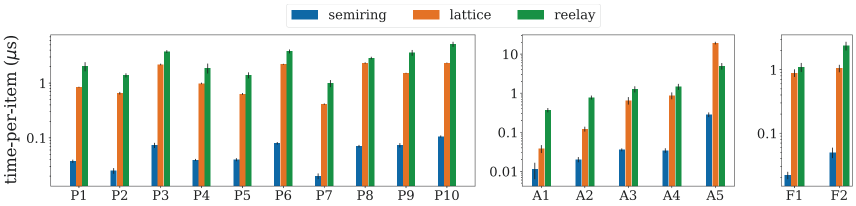

Fig. 6: Macrobenchmarks that the fuel flow rate should frequently become and remain non-zero for a sufficient amount of time. We encode this as $F_1 = \mathsf{H}_{[0,10]}\mathsf{P}_{[0,1]}(FuelFlowRate > 0)$. The other property is to ensure that whenever the air-to-fuel ratio goes out of bounds, then within 1 second it should settle back and stay there for a second. This is written as $F_2 = (\mathsf{H}_{[0,1]}airFuelRatio < 1)\ \bar{\mathsf{S}}_{[0,2]}\ airFuelRatio < 1$. The experimental results are shown in Fig. 6.

All of our experiments were executed on a laptop with an Intel Core i7 10610U CPU clocked at 2.30GHz and 16GB of memory. Each value reported is the mean of 20 executions of the experiment. The whiskers in the plots indicate the standard deviation across all executions.

### 7 Related Work

Fainekos and Pappas [22] define the robustness degree of satisfaction in terms of the distance of the signal from the set of desirable ones (or its complement). They also suggest an under-approximation of the robustness degree which can be effectively monitored. This is called the *robust semantics* and is defined by induction on STL formulas, by interpreting conjunction (resp., disjunction) as min (resp., max) of $\mathbb{R}^{\pm\infty}$. Our paper explores this robust semantics (and the related approximation guarantee) in the general algebraic setting of semirings.

In [27], the authors study a generalization of the robustness degree by considering idempotent semirings of real numbers. They also propose an online monitoring algorithm that uses symbolic weighted automata. While this approach computes the precise robustness degree in the sense of [22], the construction of the relevant automata incurs a doubly exponential blowup if one considers STL specifications. In [13], it is observed that an extension of the robust semantics to bounded distributive lattices can be effectively monitored. In this paper, we generalize this semantics by considering semirings (bounded distributive lattices are semirings). Semirings are also used in [9], where the authors consider a spatio-temporal logic. They consider the class of constraint semirings, which require the semiring order to induce a complete lattice. Efforts have been made to define notions of robustness that take temporal discrepancies into account. In [20], we see a definition of temporal robustness by considering the effect of shifting the signal in time. The "edit distance" between discretized signals is proposed as a measure of robustness in [26]. Abbas et al. [3] define a notion of $(\tau, \varepsilon)$

closeness between signals, which considers temporal and value-based guarantees separately. In [2], a metric based on conformance is put forward for applications in cardiac electrophysiology. Averaging temporal operators are used in [5], which assign a higher value to temporal obligations that are satisfied earlier.

A key ingredient for the efficient monitoring of STL is a streaming algorithm for sliding-window maximum [19,15]. The tool Breach [17,18], which is used for the falsification of temporal specifications over hybrid systems, uses the sliding-maximum algorithm of [32]. In contrast, we use a more general sliding aggregation which applies to any associative operation (not only max/min) and does not require the truth values to be totally ordered.

Different approaches for interpreting future temporal connectives in the context of online monitoring have been studied. While [16] assumes the availability of a predictor to interpret future connectives, [21] considers robustness intervals: the tightest intervals which cover the robustness for all possible extensions of the available trace prefix. Reelay [40] exclusively uses past-time connectives. The transducer-based framework of [37] can be used to monitor rich temporal properties which depend on bounded future input by allowing some bounded delay in the output.

There is a large amount of work on formalisms, domain-specific languages and associated tools for quantitative online monitoring and, more generally, for data stream processing. The synchronous language LOLA [14] has served as the basis for the StreamLAB tool [23], which is used for monitoring cyber-physical systems. Quantitative Regular Expressions [36] and associated automata-theoretic models with registers [7,8,6] have been used to express complex online detection algorithms for medical monitoring [1,4]. There are many synchronous languages and models of computation based on Kahn's dataflow model [28] that have been used for signal processing [31] and embedded controller design [12,11,10]. The construction of online monitors described in Sect. 5 relies on a set of combinators that constitute a simple domain-specific language for stream processing. Our focus here, however, is on providing efficient monitors for MTL formulas with a quantitative semantics, rather than designing a general-purpose language for monitor specification. The compositional construction of automata-based monitors from temporal specifications has also been considered in [34,35,24].

### 8 Conclusion

We have presented a new efficient algorithm for the online monitoring of MTL properties over discrete traces. We have used an abstract algebraic semantics based on semirings, which can be instantiated to the widely-used Boolean (qualitative) and robustness (quantitative) semantics, as well as to other partially ordered semirings. We also provide a theorem that relates our quantitative semantics with an algebraic generalization of the robustness degree of [22]. We have provided an implementation of our algebraic monitoring framework, and we have shown experimentally that our monitors scale reasonably well and are competitive against the state-of-the-art tool Reelay [40].

## References

[1] Abbas, H., Alur, R., Mamouras, K., Mangharam, R., Rodionova, A.: Real-time decision policies with predictable performance. Proceedings of the IEEE, Special Issue on Design Automation for Cyber-Physical Systems **106**(9), 1593–1615 (2018). 10.1109/JPROC.2018.2853608

[2] Abbas, H., Mangharam, R.: Generalized robust MTL semantics for problems in cardiac electrophysiology. In: ACC 2018. pp. 1592–1597. IEEE (2018). 10.23919/ACC.2018.8431460

[3] Abbas, H., Mittelmann, H.D., Fainekos, G.E.: Formal property verification in a conformance testing framework. In: MEMOCODE 2014. pp. 155–164. IEEE (2014). 10.1109/MEMCOD.2014.6961854

[4] Abbas, H., Rodionova, A., Mamouras, K., Bartocci, E., Smolka, S.A., Grosu, R.: Quantitative regular expressions for arrhythmia detection. IEEE/ACM Transactions on Computational Biology and Bioinformatics **16**(5), 1586–1597 (2019). 10.1109/TCBB.2018.2885274 10.1109/TCBB.2018.2885274 30530334

[5] Akazaki, T., Hasuo, I.: Time robustness in MTL and expressivity in hybrid system falsification. In: Kroening, D., Păsăreanu, C.S. (eds.) CAV 2015. LNCS, vol. 9207, pp. 356–374. Springer, Cham (2015). 10.1007/978-3-319-21668-3_21

[6] Alur, R., Fisman, D., Mamouras, K., Raghothaman, M., Stanford, C.: Streamable regular transductions. Theoretical Computer Science **807**, 15–41 (2020). 10.1016/j.tcs.2019.11.018

[7] Alur, R., Mamouras, K., Stanford, C.: Automata-based stream processing. In: ICALP 2017. Leibniz International Proceedings in Informatics (LIPIcs), vol. 80, pp. 112:1–112:15. Schloss Dagstuhl-Leibniz-Zentrum fuer Informatik, Dagstuhl, Germany (2017). 10.4230/LIPIcs.ICALP.2017.112

[8] Alur, R., Mamouras, K., Stanford, C.: Modular quantitative monitoring. Proceedings of the ACM on Programming Languages **3**(POPL), 50:1–50:31 (2019). 10.1145/3290363

[9] Bartocci, E., Bortolussi, L., Loreti, M., Nenzi, L.: Monitoring mobile and spatially distributed cyber-physical systems. In: MEMOCODE 2017. pp. 146–155. ACM (2017). 10.1145/3127041.3127050

[10] Benveniste, A., Le Guernic, P., Jacquemot, C.: Synchronous programming with events and relations: The SIGNAL language and its semantics. Science of Computer Programming **16**(2), 103–149 (1991). 10.1016/0167-6423(91)90001-E

[11] Berry, G., Gonthier, G.: The Esterel synchronous programming language: Design, semantics, implementation. Science of Computer Programming **19**(2), 87–152 (1992). 10.1016/0167-6423(92)90005-V

[12] Caspi, P., Pilaud, D., Halbwachs, N., Plaice, J.A.: LUSTRE: A declarative language for real-time programming. In: Proceedings of the 14th ACM SIGACT-SIGPLAN Symposium on Principles of Programming Languages. pp. 178–188. POPL ’87, ACM, New York, NY, USA (1987). 10.1145/41625.41641

[13] Chattopadhyay, A., Mamouras, K.: A verified online monitor for metric temporal logic with quantitative semantics. In: Deshmukh, J., Ničković, D. (eds.) RV 2020. LNCS, vol. 12399, pp. 383–403. Springer, Cham (2020). 10.1007/978-3-030-60508-7_21

[14] D’Angelo, B., Sankaranarayanan, S., Sanchez, C., Robinson, W., Finkbeiner, B., Sipma, H.B., Mehrotra, S., Manna, Z.: LOLA: Runtime monitoring of synchronous systems. In: TIME 2005. pp. 166–174. IEEE (2005). 10.1109/TIME.2005.26

[15] Deshmukh, J.V., Donzé, A., Ghosh, S., Jin, X., Juniwal, G., Seshia, S.A.: Robust online monitoring of signal temporal logic. Formal Methods in System Design **51**(1), 5–30 (2017). 10.1007/s10703-017-0286-7

[16] Dokhanchi, A., Hoxha, B., Fainekos, G.: On-line monitoring for temporal logic robustness. In: Bonakdarpour, B., Smolka, S.A. (eds.) RV 2014. LNCS, vol. 8734, pp. 231–246. Springer, Cham (2014). 10.1007/978-3-319-11164-3_19

[17] Donzé, A.: Breach, a toolbox for verification and parameter synthesis of hybrid systems. In: Touili, T., Cook, B., Jackson, P. (eds.) CAV 2010. LNCS, vol. 6174, pp. 167–170. Springer, Heidelberg (2010). 10.1007/978-3-642-14295-6_17

[18] Donzé, A.: Breach: A MATLAB toolbox for simulation-based design of dynamical/CPS/hybrid systems. https://github.com/decyphir/breach (2021), [Online; accessed January 22, 2021]

[19] Donzé, A., Ferrère, T., Maler, O.: Efficient robust monitoring for STL. In: Sharygina, N., Veith, H. (eds.) CAV 2013. LNCS, vol. 8044, pp. 264–279. Springer, Heidelberg (2013). 10.1007/978-3-642-39799-8_19

[20] Donzé, A., Maler, O.: Robust satisfaction of temporal logic over real-valued signals. In: Chatterjee, K., Henzinger, T.A. (eds.) FORMATS 2010. LNCS, vol. 6246, pp. 92–106. Springer, Heidelberg (2010). 10.1007/978-3-642-15297-9_9

[21] Dreossi, T., Dang, T., Donzé, A., Kapinski, J., Jin, X., Deshmukh, J.V.: Efficient guiding strategies for testing of temporal properties of hybrid systems. In: Havelund, K., Holzmann, G., Joshi, R. (eds.) NFM 2015. LNCS, vol. 9058, pp. 127–142. Springer, Cham (2015). 10.1007/978-3-319-17524-9_10

[22] Fainekos, G.E., Pappas, G.J.: Robustness of temporal logic specifications for continuous-time signals. Theoretical Computer Science **410**(42), 4262–4291 (2009). 10.1016/j.tcs.2009.06.021

[23] Faymonville, P., Finkbeiner, B., Schledjewski, M., Schwenger, M., Stenger, M., Tentrup, L., Torfah, H.: StreamLAB: Stream-based monitoring of cyber-physical systems. In: Dillig, I., Tasiran, S. (eds.) CAV 2019. LNCS, vol. 11561, pp. 421–431. Springer, Cham (2019). 10.1007/978-3-030-25540-4_24

[24] Ferrère, T., Maler, O., Ničković, D., Pnueli, A.: From real-time logic to timed automata. Journal of the ACM **66**(3), 19:1–19:31 (2019). 10.1145/3286976

[25] Hoxha, B., Abbas, H., Fainekos, G.E.: Benchmarks for temporal logic requirements for automotive systems. In: ARCH@CPSWeek 2014, 2015. EPiC Series in Computing, vol. 34, pp. 25–30. EasyChair (2014). 10.29007/xwrs

[26] Jakšić, S., Bartocci, E., Grosu, R., Nguyen, T., Ničković, D.: Quantitative monitoring of STL with edit distance. Formal Methods in System Design **53**(1), 83–112 (2018). 10.1007/s10703-018-0319-x 10.1007/s10703-018-0319-x PMC6428225 30956399

[27] Jakšić, S., Bartocci, E., Grosu, R., Ničković, D.: An algebraic framework for runtime verification. IEEE Transactions on Computer-Aided Design of Integrated Circuits and Systems **37**(11), 2233–2243 (2018). 10.1109/TCAD.2018.2858460

[28] Kahn, G.: The semantics of a simple language for parallel programming. Information Processing **74**, 471–475 (1974)

[29] Kong, L., Mamouras, K.: StreamQL: A query language for processing streaming time series. Proceedings of the ACM on Programming Languages **4**(OOPSLA) (2020). 10.1145/3428251

[30] Koymans, R.: Specifying real-time properties with metric temporal logic. Real-Time Systems **2**(4), 255–299 (1990). 10.1007/BF01995674

[31] Lee, E.A., Messerschmitt, D.G.: Static scheduling of synchronous data flow programs for digital signal processing. IEEE Transactions on Computers **C-36**(1), 24–35 (1987). 10.1109/TC.1987.5009446

[32] Lemire, D.: Streaming maximum-minimum filter using no more than three comparisons per element. Nordic Journal of Computing **13**(4), 328–339 (2006)

[33] Maler, O., Nickovic, D.: Monitoring temporal properties of continuous signals. In: Lakhnech, Y., Yovine, S. (eds.) FTRTFT 2004, FORMATS 2004. LNCS, vol. 3253, pp. 152–166. Springer, Heidelberg (2004). 10.1007/978-3-540-30206-3_12

[34] Maler, O., Nickovic, D., Pnueli, A.: Real time temporal logic: Past, present, future. In: Pettersson, P., Yi, W. (eds.) FORMATS 2005. LNCS, vol. 3829, pp. 2–16. Springer, Heidelberg (2005). 10.1007/11603009_2

[35] Maler, O., Nickovic, D., Pnueli, A.: From MITL to timed automata. In: Asarin, E., Bouyer, P. (eds.) FORMATS 2006. LNCS, vol. 4202, pp. 274–289. Springer, Heidelberg (2006). 10.1007/11867340_20

[36] Mamouras, K., Raghothaman, M., Alur, R., Ives, Z.G., Khanna, S.: StreamQRE: Modular specification and efficient evaluation of quantitative queries over streaming data. In: PLDI 2017. pp. 693–708. ACM (2017). 10.1145/3062341.3062369 10.1145/3140587.3062369 PMC5687100 29151821

[37] Mamouras, K., Wang, Z.: Online signal monitoring with bounded lag. IEEE Transactions on Computer-Aided Design of Integrated Circuits and Systems (2020). 10.1109/TCAD.2020.3013053

[38] The Valgrind Developers: Valgrind: An instrumentation framework for building dynamic analysis tools. https://valgrind.org/ (2021), [Online; accessed January 22, 2021]

[39] Ulus, D.: Timescales: A benchmark generator for MTL monitoring tools. In: Finkbeiner, B., Mariani, L. (eds.) RV 2019. LNCS, vol. 11757, pp. 402–412. Springer, Cham (2019). 10.1007/978-3-030-32079-9_25

[40] Ulus, D.: The Reelay monitoring tool. https://doganulus.github.io/reelay/ (2020), [Online; accessed August 20, 2020]

[41] Wolff, M.: Heaptrack: A heap memory profiler for Linux. https://github.com/KDE/heaptrack (2021), [Online; accessed January 22, 2021]

